# A Compact 2-DOF Piezoelectric-Driven Platform Based on “Z-Shaped” Flexure Hinges

**DOI:** 10.3390/mi8080245

**Published:** 2017-08-09

**Authors:** Jianping Li, Hui Liu, Hongwei Zhao

**Affiliations:** 1 School of Mechanical Science and Engineering, Jilin University, Changchun 130012, China; Jianpingli2013@gmail.com (J.L.); liuhui201707@gmail.com (H.L.); 2 Graduate School of Engineering, Chiba University, Chiba 263-8522, Japan

**Keywords:** piezoelectric, actuator, nano-positioning, flexure hinge, FEM

## Abstract

A compact 2-DOF (two degrees of freedom) piezoelectric-driven platform for 3D cellular bio-assembly systems has been proposed based on “Z-shaped” flexure hinges. Multiple linear motions with high resolution both in *x* and *y* directions are achieved. The “Z-shaped” flexure hinges and the parallel-six-connecting-rods structure are utilized to obtain the lowest working stress while compared with other types of flexure hinges. In order to achieve the optimized structure, matrix-based compliance modeling (MCM) method and finite element method (FEM) are used to evaluate both the static and dynamic performances of the proposed 2-DOF piezoelectric-driven platform. Experimental results indicate that the maximum motion displacements for *x*-stage and *y*-stage are *l*_x_ = 17.65 μm and *l*_y_ = 15.45 μm, respectively. The step response time for *x*-stage and *y*-stage are *t*_x_ = 1.7 ms and *t*_y_ = 1.6 ms, respectively.

## 1. Introduction

The 3D cellular bio-assembly system has become a hot research topic during recent years. In order to manipulate cells and biological tissues in 3D space, micro/nano-positioning systems with high resolution and multi-DOF (multiple degrees of freedom) should be utilized in 3D cellular bio-assembly systems [1]. 

Piezoelectric-driven multi-DOF platforms are widely utilized in 3D cellular bio-assembly systems for their own advantages: high speed, simple control, high resolution, and multiple degrees of freedom [2,3]. Up to now, several kinds of piezoelectric-driven multi-DOF platforms have been developed, and they are mainly divided into two types: parallel-type and serial-type. In the parallel-type piezoelectric-driven multi-DOF platform, all piezoelectric elements drive only one moving platform to obtain multi-DOF motions, which reduces the whole size and the inertia simultaneously [4,5,6,7]. Additionally, the dynamic performance and orthogonality are generally better than serial type ones. However, in order to reap the merits of parallel-type piezoelectric-driven platforms, direct drivetrain-output metrology in all the motion directions with high resolution should be carried out carefully. Some corporations, such as *PI*, *Attocube* and *SMartAct*, have already developed the commercial parallel-type piezoelectric-driven multi-DOF platforms [8,9,10], they utilize several positioning sensors to monitor all the motions to reduce the coupling errors, which greatly brings much high cost and complicated control systems. Compared with the parallel-type, serial-type piezoelectric-driven platforms can realize small coupling errors even with open-loop control systems. The dynamic performance of serial-type platforms is not as good as the parallel-type platforms, but serial-type platforms are quite suitable for the application with low cost, easy control systems and high reliability, where the requirement for dynamic performance is not so high. Generally, serial-type piezoelectric-driven platforms are divided into two forms: stacked form and nested form. In the stacked form platforms, one layer only achieves one degree of freedom. Several motion principles have been applied to obtain this only one motion degree of freedom, for instance stick-slip principle [11,12,13], inchworm principle [14,15,16,17] and ultrasonic principle [18,19,20]. Afterwards, several layers should be stacked together to obtain the multi-DOF motion, which causes the challenge of reliability because of abrasion and complicated structures. Meanwhile, it also greatly limits the application in 3D cellular bio-assembly systems [8,21,22,23]. To overcome these shortcomings, one or more motion degrees of freedom are nested inside one basic motion to achieve the nested form platforms [24,25,26]. Nevertheless, most of the previous nested form piezoelectric-driven platforms utilize the basic right-angle and right-circle flexure hinges, which causes great structural stress and influences the whole working lifetime. 

In 3D cellular bio-assembly systems, the size of tissue cells is almost around 10 μm, which means that the positioning platform with submicron accuracy and multiple degrees of freedom is enough for the cell manipulation. The working frequency needs not to be too high. Additionally, high reliability is also required, since the 3D cellular bio-assembly process always takes a long time for thousands of cells or more. Hence, the nested form serial-type piezoelectric-driven platform is quite suitable for 3D cellular bio-assembly systems to achieve the submicron accuracy, simple structure and control system, and low cost. In order to achieve high reliability and long service lifetime, the used flexure hinges for piezoelectric platforms should have low working stress. However, the most utilized right-angle and right-circle flexure hinges always have high working stress, which greatly influences the working lifetime and the reliability. Therefore, it is of great significance to develop the piezoelectric-driven platforms with lower stress on the flexure hinges.

In this study, in order to reduce the working stress of flexure hinges and obtain multiple degrees of freedom motions for 3D cellular bio-assembly systems with high reliability, a compact serial-type piezoelectric-driven platform based on “Z-shaped’’ flexure hinges is proposed and manufactured. The “Z-shaped’’ flexure hinge is compared with right-angle and right-circle flexure hinges by matrix-based compliance modeling (MCM) method and finite element method (FEM) to confirm it has the lowest working stress under the same condition. The parallel-six-connecting-rods structure is optimized by FEM method to enhance platform stiffness and make the platform stable. The experimental system is established to test the performance of the proposed two degrees of freedom (2-DOF) piezoelectric-driven platform. Additionally, the open-loop control method with respect to the coupling error is developed to achieve high positioning resolution.

## 2. Materials and Methods 

### 2.1. Calculation of the “Z-shaped” Flexure Hinge

Flexure hinges are widely exploited in piezoelectric-driven platforms, due to their unique superiorities: high resolution, compact size, no friction and simple structure. Several kinds of flexure hinges have been developed for different applications. Among them, the right-angle flexure hinge and right-circle flexure hinge are the most used ones. However, during the application of right-angle and right-circle flexure hinges, the working stress is always high, which greatly influences the working lifetime of the whole piezoelectric-driven platform. Compared with other kinds of flexure hinges, the “Z-shaped” flexure hinge achieves larger output displacement with smallest working stress, which is ideally qualified for the piezoelectric-driven platform with long working lifetime.

**Figure 1:**
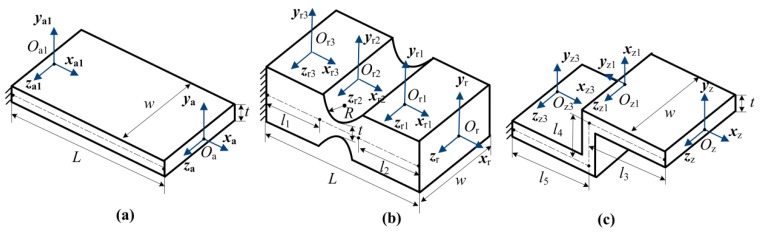
Structure of three kinds of flexure hinges: (**a**) right-angle; (**b**) right-circle; (**c**) “Z-shaped”.

In order to compare the working stress of right-angle, right-circle and “Z-shaped” flexure hinges under the same condition, matrix-based compliance modeling (MCM) method is applied for the theoretical calculation. Figure 1 shows the basic structure parameters of the right-angle, right-circle and “Z-shaped” flexure hinges, and they are treated as the integration of basic flexure hinges (right-angle and right-circle). As is shown in Figure 1a, the right-angle flexure hinge is a basic flexure hinge, which is only made up by one part *O*_a_*O*_a1_; the structure is *L* = 16 mm, *w* = 6 mm and *t* = 1 mm. Figure 1b illustrates the right-circle flexure hinge, and it is divided into three parts: part *O*_r_*O*_r1_ (basic right-angle), part *O*_r1_*O*_r2_ (basic right-circle) and part *O*_r2_*O*_r3_ (basic right-angle); the structure is *l*_1_ = *l*_2_ = 5 mm, *R* = 3 mm. The “Z-shaped” flexure hinge is shown in Figure 1c, and it consists of three basic right-angle flexure hinges: part *O*_z_*O*_z1_, part *O*_z1_*O*_z2_ and part *O*_z2_*O*_z3_; the size is *l*_3_ = *l*_5_ = 8 mm, *l*_4_ = 6 mm. According to the references [27,28], the widely utilized compliance matrix *C*_ra_ for the right-angle flexure hinge is gotten by the following:

(1) $$C_{\mathrm{ra}}=\left[\begin{array}{cccccc} \frac{L}{Ewt} & 0 & 0 & 0 & 0 & 0\\ 0 & \frac{4L^{3}}{Ewt^{3}}+\frac{L}{Gwt} & 0 & 0 & 0 & \frac{6L^{2}}{Ewt^{3}}\\ 0 & 0 & \frac{4L^{3}}{Ew^{3}t}+\frac{L}{Gwt} & 0 & -\frac{6L^{2}}{Ew^{3}t} & 0\\ 0 & 0 & 0 & \frac{L}{Gkwt^{3}} & 0 & 0\\ 0 & 0 & -\frac{6L^{2}}{Ew^{3}t} & 0 & \frac{12L}{Ew^{3}t} & 0\\ 0 & \frac{6L^{2}}{Ewt^{3}} & 0 & 0 & 0 & \frac{12L}{Ewt^{3}} \end{array}\right]$$

where *E* is the elastic module of the applied material; *G* is the shear module; *L*, *w*, *t* are the structural parameters of the basic right-angle flexure hinge.

For the basic right-circle flexure hinge, the compliance matrix *C*_rc_ is shown in the following [29]:

(2) $$C_{\mathrm{rc}}=\left[\begin{array}{cccccc} \frac{du_{x}}{dF_{x}} & 0 & 0 & 0 & 0 & 0\\ 0 & \frac{du_{y}}{dF_{y}} & 0 & 0 & 0 & \frac{du_{y}}{dM_{z}}\\ 0 & 0 & \frac{du_{z}}{dF_{z}} & 0 & \frac{du_{z}}{dM_{y}} & 0\\ 0 & 0 & 0 & \frac{d\theta_{x}}{dM_{x}} & 0 & 0\\ 0 & 0 & \frac{d\theta_{y}}{dF_{z}} & 0 & \frac{d\theta_{y}}{dM_{y}} & 0\\ 0 & \frac{d\theta_{z}}{dF_{y}} & 0 & 0 & 0 & \frac{d\theta_{z}}{dM_{z}} \end{array}\right]$$

Define *g* = *R*/*t*:

(3) $$\frac{du_{x}}{dF_{x}}=\frac{1}{Ew}\left[\frac{2\left(2g+1\right)}{\sqrt{4g+1}}\arctan\sqrt{4g+1}-\frac{\pi }{2}\right]$$

(4) $$\frac{du_{y}}{dF_{y}}={\left(\frac{du_{y}}{dF_{y}}\right)}_{\sigma }+{\left(\frac{du_{y}}{dF_{y}}\right)}_{\tau }$$

(5) $${\left(\frac{du_{y}}{dF_{y}}\right)}_{\sigma }=\frac{12}{Ew}\left[\begin{array}{l} \frac{g\left(24g^{4}+24g^{3}+22g^{2}+8g+1\right)}{2\left(2g+1\right){\left(4g+1\right)}^{2}}+\\ \frac{\left(2g+1\right)\left(24g^{4}+8g^{3}-14g^{2}-8g-1\right)}{2{\left(4g+1\right)}^{2.5}}\arctan\sqrt{4g+1}+\frac{\pi }{8} \end{array}\right]$$

(6) $${\left(\frac{du_{y}}{dF_{y}}\right)}_{\tau }=\frac{1}{Gw}\left[\frac{2\left(2g+1\right)}{\sqrt{4g+1}}\arctan\sqrt{4g+1}-\frac{\pi }{2}\right]$$

(7) $$\frac{du_{z}}{dF_{z}}={\left(\frac{du_{z}}{dF_{z}}\right)}_{\sigma }+{\left(\frac{du_{z}}{dF_{z}}\right)}_{\tau }$$

(8) $${\left(\frac{du_{z}}{dF_{z}}\right)}_{\sigma }=\frac{12R^{2}}{Ew^{3}}\left[\begin{array}{l} \frac{2g+1}{2g}+\frac{\left(2g+1)(4g^{2}-4g-1\right)}{2g^{2}\sqrt{4g+1}}\arctan\sqrt{4g+1}\\ -\frac{2g^{2}-4g-1}{8g^{2}}\pi \end{array}\right]$$

(9) $${\left(\frac{du_{z}}{dF_{z}}\right)}_{\tau }=\frac{1}{Gw}\left[\frac{2\left(2g+1\right)}{\sqrt{4g+1}}\arctan\sqrt{4g+1}-\frac{\pi }{2}\right]$$

(10) $$\frac{du_{y}}{dM_{z}}=-\frac{12}{EwR}\left[\frac{2g^{3}\left(6g^{2}+4g+1\right)}{\left(2g+1\right){\left(4g+1\right)}^{2}}+\frac{12g^{4}\left(2g+1\right)}{{\left(4g+1\right)}^{2.5}}\arctan\sqrt{4g+1}\right]$$

where *u*_x_, *u*_y_ and *u*_z_ are the linear deformation along the *x*, *y* and *z* directions; *θ*_x_, *θ*_y_ and *θ*_z_ are the rotational angles around the *x*, *y* and *z* directions; *F*_x_, *F*_y_, *F*_z_, *M*_x_, *M*_y_ and *M*_z_ are the applied forces and torques in the *x*, *y* and *z* directions.

Since the right-angle flexure hinge shown in Figure 1a is a basic flexure hinge, the total compliance matrix $C_{\mathrm{ra}}^{f}$ of it is as the following:

(11) $$C_{\mathrm{ra}}^{f}=C_{\mathrm{ra}}$$

The right-circle flexure hinge is divided into three parts (Figure 1b), and it is treated as the series structure of three basic flexure hinges according to the MCM method. Hence, the total compliance matrix $C_{\mathrm{rc}}^{f}$ is obtained by:

(12) $$C_{\mathrm{rc}}^{f}=C_{\mathrm{or}}^{f}+C_{\mathrm{or}1}^{f}+C_{\mathrm{or}2}^{f}=C_{\mathrm{ra}}^{}+T_{\mathrm{or}1}^{f}C_{\mathrm{rc}}^{}{\left(T_{\mathrm{or}1}^{f}\right)}^{T}+T_{\mathrm{or}2}^{f}C_{\mathrm{ra}}^{}{\left(T_{\mathrm{or}2}^{f}\right)}^{T}$$

where $T_{\mathrm{or}1}^{f}$ and $T_{\mathrm{or}2}^{f}$ are the transform matrixes of point *O*_ri_ with respect to point *O*_r_, which are achieved by:

(13) $$T_{\mathrm{ori}}^{f}=\left[\begin{array}{cc} R_{\mathrm{ori}}(\alpha_{\mathrm{ori}}) & S_{\mathrm{ori}}R_{\mathrm{ori}}(\alpha_{\mathrm{ori}})\\ 0 & R_{\mathrm{ori}}(\alpha_{\mathrm{ori}}) \end{array}\right],\;{}^{}(i=1,2)$$

(14) $$R_{\mathrm{ori}}(\alpha_{\mathrm{ori}})=\left[\begin{array}{ccc} \cos\alpha_{\mathrm{ori}} & \sin\alpha_{\mathrm{ori}} & 0\\ -\sin\alpha_{\mathrm{ori}} & \cos\alpha_{\mathrm{ori}} & 0\\ 0 & 0 & 1 \end{array}\right],\;{}^{}(i=1,2)$$

(15) $$S_{\mathrm{ori}}=\left[\begin{array}{ccc} 0 & -r_{\mathrm{zri}} & r_{\mathrm{yri}}\\ r_{\mathrm{zri}} & 0 & r_{\mathrm{xri}}\\ -r_{\mathrm{yri}} & r_{\mathrm{xri}} & 0 \end{array}\right],\;{}^{}(i=1,2)$$

where *R*_ori_(*α*_ori_) and *S*_ori_ are the rotation matrix and position matrix of point *O*_ri_ with respect to point *O*_r_, respectively; *α*_ori_ is the rotation angle of point *O*_ri_ relative to point *O*_r_; *r*_xri_, *r*_yri_ and *r*_zri_ are the relative positions between point *O*_ri_ and point *O*_r_. 

The developed “Z-shaped” consists of three basic right-angle flexure hinges (Figure 1c), and the total compliance matrix $C_{z}^{f}$ is obtained by the following:

(16) $$C_{z}^{f}=C_{\mathrm{oz}}^{f}+C_{\mathrm{oz}1}^{f}+C_{\mathrm{oz}2}^{f}=C_{\mathrm{ra}}^{}+T_{\mathrm{oz}1}^{f}C_{\mathrm{ra}}^{}{\left(T_{\mathrm{oz}1}^{f}\right)}^{T}+T_{\mathrm{oz}2}^{f}C_{\mathrm{ra}}^{}{\left(T_{\mathrm{oz}2}^{f}\right)}^{T}$$

(17) $$T_{\mathrm{ozi}}^{f}=\left[\begin{array}{cc} R_{\mathrm{ozi}}(\alpha_{\mathrm{ozi}}) & S_{\mathrm{ozi}}R_{\mathrm{ozi}}(\alpha_{\mathrm{ozi}})\\ 0 & R_{\mathrm{ozi}}(\alpha_{\mathrm{ozi}}) \end{array}\right],\;{}^{}(i=1,2)$$

(18) $$R_{\mathrm{ozi}}(\alpha_{\mathrm{ozi}})=\left[\begin{array}{ccc} \cos\alpha_{\mathrm{ozi}} & \sin\alpha_{\mathrm{ozi}} & 0\\ -\sin\alpha_{\mathrm{ozi}} & \cos\alpha_{\mathrm{ozi}} & 0\\ 0 & 0 & 1 \end{array}\right],\;{}^{}(i=1,2)$$

(19) $$S_{\mathrm{ozi}}=\left[\begin{array}{ccc} 0 & -r_{\mathrm{zzi}} & r_{\mathrm{yzi}}\\ r_{\mathrm{zzi}} & 0 & r_{\mathrm{xzi}}\\ -r_{\mathrm{yzi}} & r_{\mathrm{xzi}} & 0 \end{array}\right],\;{}^{}(i=1,2)$$

where $T_{\mathrm{oz}1}^{f}$ and $T_{\mathrm{or}2}^{f}$ are the transform matrixes of point *O*_zi_ with respect to point *O*_z_; *R*_ozi_(*α*_ozi_) and *S*_ozi_ are the rotation matrix and position matrix of point *O*_zi_ to point *O*_z_, respectively. 

Therefore, the total compliance matrixes of the right-angle, right-circle and “Z-shaped” flexure hinge are achieved by the Equations (11), (12) and (16), respectively.

In order to confirm the theoretical calculation by MCM method, FEM has been applied to simulate the deformation of these three flexure hinges. Three kinds of flexure hinge (right-angle, right-circle and “Z-shaped”) have been calculated by FEM, and the material of them is steel. The elastic module is set as *E* = 206 GPa, and the Poisson ratio is *μ* = 0.288 during the FEM simulation. The structural parameters are the same with those shown in Figure 1. During the FEM calculation, the left end of each flexure hinge is fixed to the ground without freedom. An input force *F* = 20 N along *y* direction is applied at the right end surface of each flexure hinge. The FEM mesh size is 0.1 mm for these three flexure hinges.

**Figure 2:**
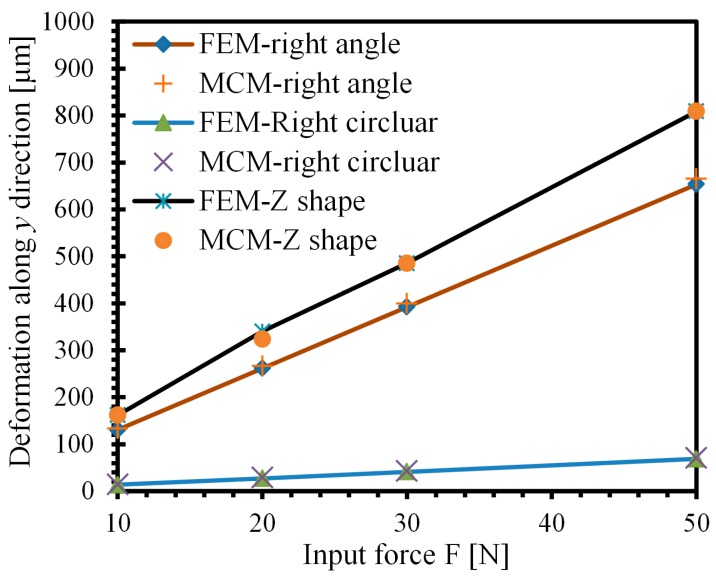
Comparison of FEM and MCM results for three kinds of flexure hinges.

**Figure 3:**
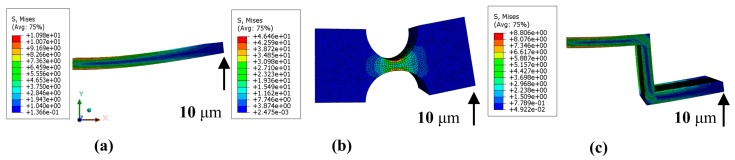
FEM results of different flexure hinges: (**a**) right-angle; (**b**) right-circle; (**c**) “Z-shaped”.

The deformation results both from the MCM and FEM methods are shown in Table 1. The maximum deformation of the “Z-shaped” flexure hinge along *y* direction is *D*_z_ = 323.5 μm by the MCM method, while that from the FEM method is *D*_z’_ = 340.1 μm; the error between the MCM and FEM methods is *e*_z_ = 4.9% based on Equation (20), which confirms the application feasibility of the MCM method. The maximum deformation for the right-angle flexure hinge is *D*_ra_ = 265.8 μm with an error of *e*_ra_ = 1.7%. The error of the right-circle flexure hinge is *e*_rc_ = 2.2%, considering the influence of the torque induced by input force *F* in Equation (10). The comparison of FEM and MCM methods under other forces can be obtained in Figure 2, which shows the great agreement. Figure 3 illustrates the working stress of these three flexure hinges by FEM method in the case that a displacement *D*_y_ along *y* direction is applied on the right end surface of each flexure hinge. The left end of each flexure hinge is fixed to the ground without freedom. The FEM mesh size is 0.1 mm for these three flexure hinges. Since the working stroke of the utilized piezo stack is around 10 μm while the driving voltage is 100 V, the applied displacement is chosen as *D*_y_ = 10 μm. It is shown that the maximum working stress of these three flexure hinges are *σ*_ra_ = 10.9 MPa, *σ*_rc_ = 46.5 MPa and *σ*_z_ = 8.8 MPa. The working stress of the “Z-shaped” flexure hinge is the smallest among them, which is of great significance to extend the working lifetime of piezoelectric-driven platforms. Hence, the “Z-shaped” flexure hinge is utilized for the design of the proposed 2-DOF nano-positioning platform.

(20) $$e_{z}=\frac{\left|D_{z}-D_{z^{\prime }}\right|}{D_{z^{\prime }}}\times 100\%$$

### 2.2. Platform Design

**Figure 4:**
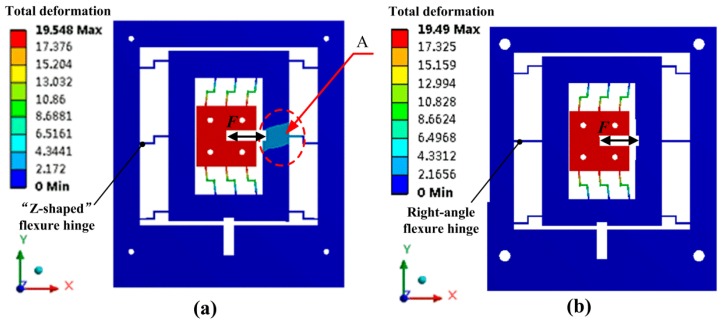
FEM static results of the platform: (**a**) only “Z-shaped” flexure hinges; (**b**) with right-angle flexure hinges.

Based on “Z-shaped” flexure hinges, a 2-DOF piezoelectric-driven platform has been proposed and manufactured. The parallel-six-connecting-rods structure is applied both in *x*-stage and *y*-stage instead of the generally used parallel-four-connecting-rods, and it is helpful to enhance the output stiffness of the whole platform. Additionally, it makes the platform more stable and the coupling error smaller, since the stiffness of *x*- and *y*-stages is enhanced to reduce coupling deformation of reaction forces. FEM method is also used to calculate the performance of the proposed 2-DOF piezoelectric-driven platform. During the FEM calculation, the four holes on the base corners are fixed to the ground without motion degrees of freedom; an input force *F* = 50 N was applied on *x*-stage in the surface to nest the piezoelectric stack; meanwhile, the reaction force *F*_r_ = 50 N was also applied on the surface to nest the piezoelectric stack on *y*-stage. Figure 4 illustrates the results of FEM calculation. In the case that all the six-connecting-rods for *y*-stage are “Z-shaped” flexure hinges, the deformation of area A in *y*-stage is about 3 μm, as shown in Figure 4a. Since the piezoelectric stack for *x* direction is nested inside *y*-stage, the reaction force makes the *y*-stage generate this unwanted deformation, which should be avoided. Meanwhile, in the case that two right-angle flexure hinges are utilized to replace “Z-shaped” flexure hinges for *y*-stage, the unwanted deformation of area A is rather small since the stiffness of right-angle flexure hinge along *x* direction is higher than the “Z-shaped” flexure hinge. Additionally, the output force of the used piezoelectric stacks is around *F*_stack_ = 800 N, which is large enough to reduce the influence of the increased stress for *y*-stage motion stroke. Therefore, both “Z-shaped” flexure hinges and right-angle flexure hinges are utilized in the 2-DOF piezoelectric-driven platform. 

**Figure 5:**
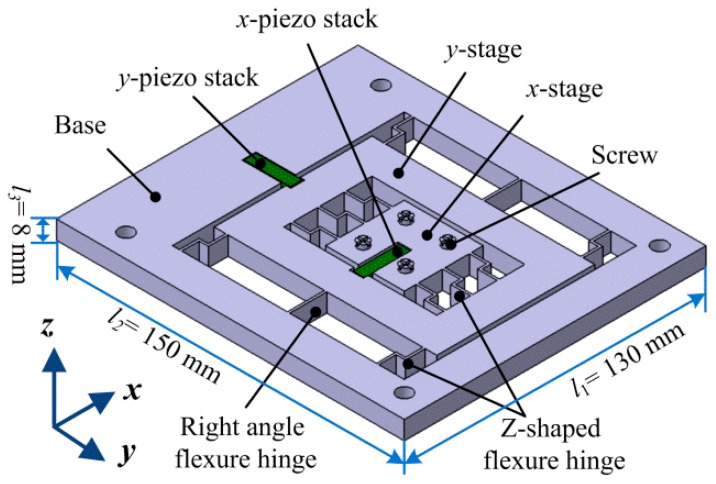
Structure of the proposed 2-DOF (two degrees of freedom) piezoelectric-driven platform.

Figure 5 shows the proposed 2-DOF piezoelectric-driven platform which consists of two piezoelectric stacks (*x*-piezo stack and *y*-piezo stack), *x*-stage, *y*-stage, screws, “Z-shaped” flexure hinges, right-angle flexure hinges and the base. The *x*-piezo stack is used to drive *x*-stage; *y*-piezo stack is applied for *y*-stage. These two piezo stacks (AE0505D16, 5 mm × 5 mm × 20 mm) are from NEC COMPANY; the maximum output force of them is around *F*_stack_ = 800 N, and the motion stroke is about *L*_stack_ = 17 ± 2 μm in the case of input voltage *V* = 150 V; they are working on the *d*_33_ model and the value is *d*_33_ = 720 × 10^−12^ m/V. The *x*-stage is nested inside of *y*-stage, which means that the proposed nano-positioning platform is in serial type. This serial structure can make the proposed platform in a compact size and with small coupling errors. Two kinds of flexure hinges (“Z-shaped” and right-angle) are exploited to form a parallel-six-connecting-rods structure for *y*-stage; the flexure hinges in *x*-stage are all “Z-shaped” flexure hinges. The material of the proposed 2-DOF piezoelectric-driven platform is structural steel. Wire electrical discharge machining (WEDM) was utilized to manufacture the platform, so as to make the whole platform in an integrated structure. The size of the proposed 2-DOF piezoelectric-driven platform is *l*_1_ × *l*_2_ × *l*_3_ = 130 mm × 150 mm × 8mm.

### 2.3. Dynamic Calculation

**Figure 6:**
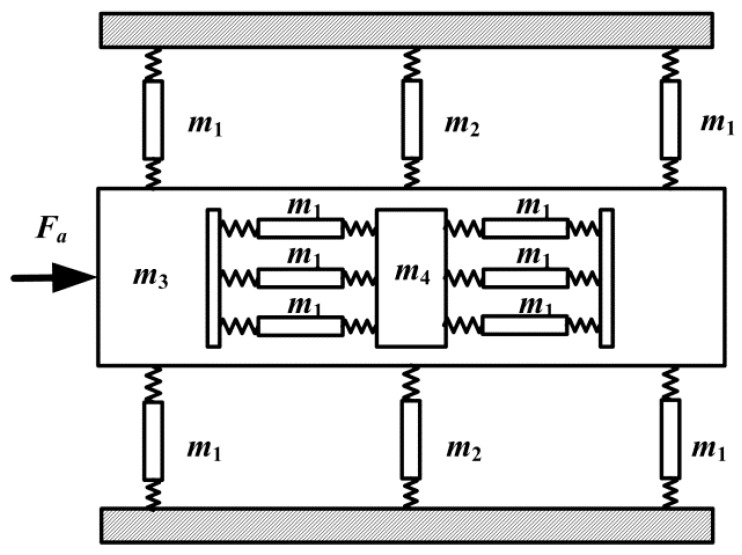
Dynamic model of the proposed 2-DOF piezoelectric-driven platform.

Figure 6 illustrates the dynamic model of the proposed 2-DOF piezoelectric-driven platform. To simplify the calculation, all parts of the platform are treated as rigid bodies, except the flexure hinges. According to the law of energy conservation, the total energy value of kinetic energy *E*_k_ and potential energy *E*_t_ keeps the same during the motion. In the case that force *F*_a_ is applied on *y*-stage, the platform moves a distance *s*. Kinetic energy *E*_k_ of the proposed platform is divided into two parts: kinetic energy *E*_k1_ induced by the linear motion, kinetic energy *E*_k2_ induced by the rotation motion of flexure hinges. Kinetic energy *E*_k_ is achieved by the following equation [27]:

(21) $$E_{K1}=\frac{1}{2}(m_{3}+m_{4}+6m_{1})s^{\prime }{}^{2}$$

(22) $$E_{K}{}_{2}=4\times \frac{1}{2}J_{Z}\theta^{\prime }{}^{2}+2\times \frac{1}{2}J_{r}\theta^{\prime }{}^{2}=2J_{Z}\theta^{\prime }{}^{2}+J_{r}\theta^{\prime }{}^{2}$$

(23) $$E_{K}=E_{K1}+E_{K2}=\frac{1}{2}(m_{3}+m_{4}+6m_{1})s^{\prime }{}^{2}+2J_{Z}\theta^{\prime }{}^{2}+J_{r}\theta^{\prime }{}^{2}$$

where *m*_1_ is the mass of the “Z-shaped” flexure hinge; *m*_2_ is the mass of the right-angle flexure hinge; *m*_3_ is the mass of *y*-stage; *m*_4_ is the mass of *x*-stage; *s* is the motion displacement of *y*-stage; *θ* = arctan (*s*/*L*) ≈ *s*/*L* is the rotation angular displacement of flexure hinge; *J*_z_ is the inertia moment of the “Z-shaped” flexure hinge; *J*_r_ is the inertia moment of the right-angle flexure hinge. 

Potential energy *E*_t_ is mainly induced by the six flexure hinges for *y*-stage, and it is obtained by the following equation:

(24) $$E_{t}=\int_{0}^{\theta }(4k_{\theta z}+2k_{\theta r})xdx=(2k_{\theta z}+k_{\theta r})\theta^{2}$$

where *k*_θz_ is the rotational stiffness of the “Z-shaped” flexure hinge; *k*_θr_ is the rotational stiffness of the right-angle flexure hinge. 

Therefore, the total energy *E*_0_ keeps the same and it can be achieved by the following equation:

(25) $$E_{0}=E_{k}+E_{t}=\frac{1}{2}(m_{3}+m_{4}+6m_{1})s^{\prime }{}^{2}+2J_{z}\theta^{\prime }{}^{2}+J_{r}\theta^{\prime }{}^{2}+(2k_{\theta z}+k_{\theta r})\theta^{2}$$

However, it is difficult to get the accurate solution of Equation (25). Hence, FEM method is applied to analyze the dynamic performance of the proposed 2-DOF piezoelectric-driven platform. FEM dynamic analysis results are obtained in Table 2. The resonance frequencies from the first dynamic modal to the sixth dynamic modal are *f*_1_ = 678 Hz, *f*_2_ = 965 Hz, *f*_3_ = 1297 Hz, *f*_4_ = 1553 Hz, *f*_5_ = 1832 Hz and *f*_6_ = 2002 Hz. It can be seen that the undesired transverse motion (*z* and *θ*_x_) is introduced in high frequency since the use of “Z-shaped” flexure hinges, which is not good for the 2-DOF nano-positioning platform. Generally, the proposed 2-DOF nano-positioning platform works under several dozens of Hertz; hence, the resonance phenomenon could be avoided. We are still working on this platform, and hope to overcome this disadvantage in future work.

## 3. Results and Discussion

### 3.1. Experimental System

**Figure 7:**
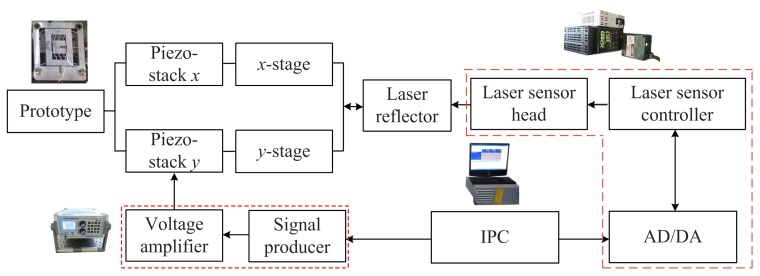
Experimental system for the proposed 2-DOF piezoelectric-driven platform.

Figure 7 shows the experimental system for the proposed 2-DOF piezoelectric-driven platform. It is composed by one signal producer with the inserted voltage amplifier, one laser sensor with laser head and controller, the laser reflector, the prototype with piezo-stacks (*x*, *y*) and one industrial personal computer (IPC). The signal producer (HPV, BOSHI COMPANY) generates the needed voltage signal for the piezo-stacks, and the voltage signal is amplified by the voltage amplifier (integrated inside of HPV, BOSHI COMPANY) to a high value to drive the piezo-stacks in *x*- and *y*-stages. The maximum output voltage of this HPV is *U*_HPV_ = ±150 V with a voltage resolution of *e*_HPV_ = 1%; the maximum output current is *I*_HPV_ = 0.5 A. The laser reflectors are attached to the *x*- and *y*- stages to reflect the laser signal. Laser sensor LK-G10 (KEYENCE COMPANY) is utilized to generate the laser signal to record the motion of the laser reflector. The laser head is placed in line with the motion direction of *x*-/*y*- stage which has the attached laser reflector. The measurement range of LK-G10 is *L*_LK_ = ±1 mm with a resolution of *e*_LK_ = 10 nm which is high enough for the proposed piezoelectric-driven platform. All data is processed and changed by the AD/DA card which is integrated inside the laser sensor LK-G10, and then saved by the IPC. The commercial software from KEYENCE COMPANY is utilized to control the laser sensor and save the data with a sampling time of *t*_sa_ = 0.1 ms. The software from BOSHI COMPANY is exploited to control the signal producer. All this equipment is placed on the air-floating vibration isolation platform to isolate the vibration out of the experimental system.

### 3.2. Output Performance

In the real application, the proposed piezoelectric-driven platform is fixed to a motor-driven 3-DOF platform to obtain enough motion strokes. This motor-driven platform is composed of three motors, three ball screws and linear guiders to obtain large working stroke in *x*, *y* and *z* directions (80 mm × 80 mm × 120 mm). However, the 3-DOF motor-driven platform is a traditional one, not the originality of our paper, and the resolution is not high enough for cell manipulation; hence, it is not mentioned in our paper. Here, the shown experiments are for the proposed 2-DOF piezoelectric-driven platform.

(a) Motion performance in *x* direction

**Figure 8:**
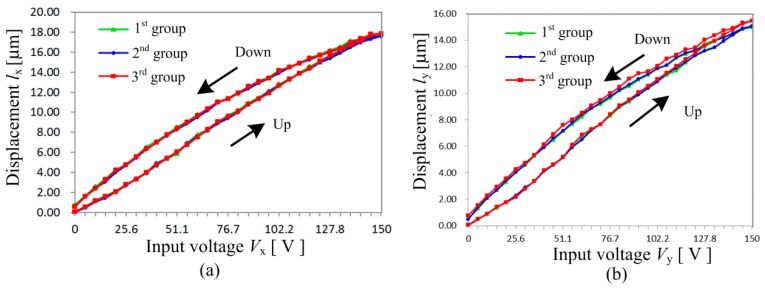
Motion performance: (**a**) *x*-stage; (**b**) *y*-stage.

The motion performance of *x*-stage under different input voltage *V*_x_ is illustrated in Figure 8a. The input voltage *V*_x_ goes up from *V*_x_ = 0 V to *V*_x_ = 150 V, and then falls down to *V*_x_ = 0 V. The motion displacement *l*_x_ of *x*-stage increases to *l*_x_ = 17.65 μm in the case of input voltage *V*_x_ = 150 V. This experiment is repeated three times, and the repeatability is found excellent. The maximum displacement error between the loading (up) and unloading (down) curves is *e*_x_ = 0.36 μm, which is thought to be induced by the hysteresis and system errors. Compared with the maximum motion displacement, the maximum relative displacement error is *e*_xr_ = 2.03%. Many researchers have paid attention to reduce the hysteresis of piezoelectric actuators by closed-loop control systems and compensation algorithms. However, these methods make the whole system complicated and very costly. The requirement of the resolution in 3D cellular bio-assembly systems is not so high, since the size of cells is almost around 10 μm. Therefore, the influence of hysteresis is not discussed deeply here.

(b) Motion performance in *y* direction

Figure 8b shows the motion performance of *y*-stage under different input voltage *V*_y_. The input voltage *V*_y_ also increases from *V*_y_ = 0 V to *V*_y_ = 150 V, and then decreases to *V*_y_ = 0 V. The maximum motion displacement of *y*-stage is *l*_y_ = 15.45 μm in the case that the input voltage *V*_y_ = 150 V. This experiment is also repeated three times, and the maximum displacement error between the loading (up) and unloading (down) curves is *e*_y_ = 0.56 μm, which is also thought to be induced by the hysteresis and system errors. Hence, the maximum relative displacement error is about *e*_yr_ = 3.62%, which is a little larger than that in *x*-stage. The difference between the repeated three groups is thought to be caused by the assembly errors of the piezoelectric stacks and the system errors. 

(c) Displacement coupling error

**Figure 9:**
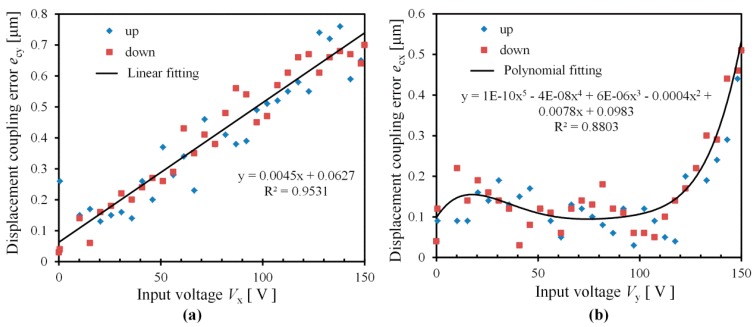
Displacement coupling errors: (**a**) coupling error for *y*-stage; (**b**) coupling error for *x*-tage.

The displacement coupling errors *e*_cy_ and *e*_cx_ are achieved from Figure 9. In the case that the input voltage *V*_x_ is applied to the *x* piezo stack, the laser sensor is utilized to measure the displacement of *y*-stage in *y* direction. Hence, the displacement coupling error *e*_cy_ is shown in Figure 9a. It can be seen that the displacement coupling error *e*_cy_ increases with input voltage *V*_x_. The maximum displacement coupling error for *y*-stage is *e*_cy_ = 0.76 μm, in the case that the input voltage *V*_x_ = 138 V. The relationship between the input voltage *V*_x_ and the displacement coupling error *e*_cy_ is simplified by the following equation with a correlation coefficient of *R*^2^ = 0.95:

(26) $$e_{\mathrm{cy}}=0.0045V_{x}+0.0627$$

Similarly, the displacement coupling error *e*_cx_ for *x*-stage is obtained in the case that input voltage *V*_y_ is applied for *y*-piezo stack (Figure 9b). The maximum displacement error *e*_cx_ for *x*-stage is *e*_cx_ = 0.51 μm, in the case of input voltage *V*_y_ = 150 V. The relationship between *e*_cx_ and *V*_y_ is nonlinear by the polynomial equation:

(27) $$e_{\mathrm{cx}}=10^{-10}V_{x}{}^{5}-4\times 10^{-8}V_{x}{}^{4}+6\times 10^{-6}V_{x}{}^{3}-0.0004V_{x}{}^{2}+\;0.0078V_{x}\;+\;0.0983$$

(d) Output force

**Figure 10:**
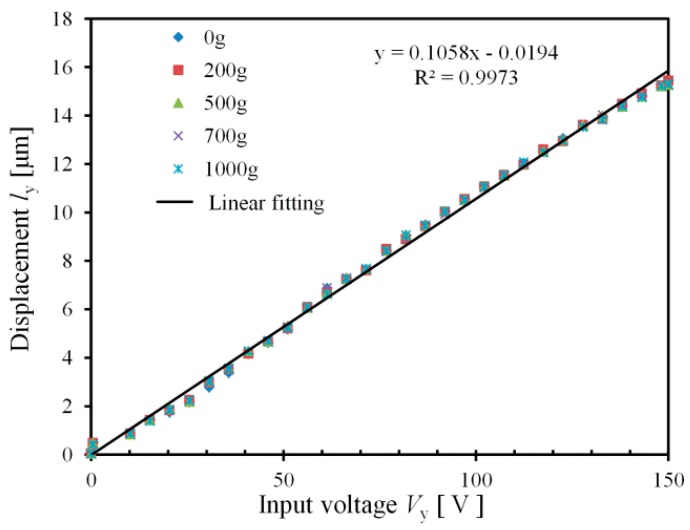
Performance of proposed 2-DOF platform under different output force.

The performance of the proposed 2-DOF piezoelectric-driven platform under different output force has been measured, as shown in Figure 10. Stand weight was put on *x*-stage during the measurement. The motion displacement *l*_y_ of *y*-stage keeps almost the same, in the case that stand weight increases from *m* = 0 g to *m* = 1000 g, which indicates that the proposed 2-DOF piezoelectric-driven platform works stably with high output force.

(e) Step response

**Figure 11:**
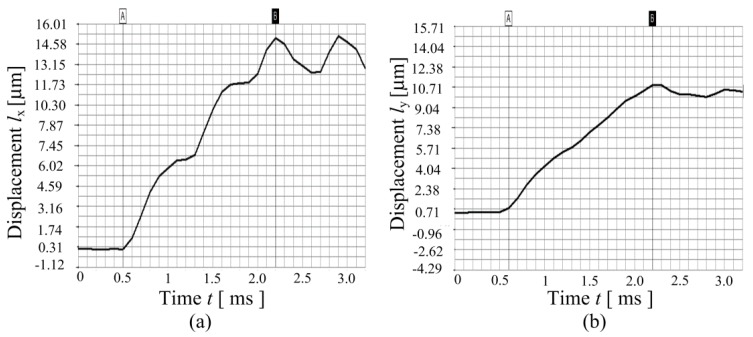
Step response of the proposed 2-DOF platform: (**a**) *x*-stage; (**b**) *y*-stage.

Figure 11 illustrates the step response of the proposed 2-DOF piezoelectric-driven platform. A plus voltage signal with the amplitude of 100 V is applied for both *x*-piezo stack and *y*-piezo stack. The sampling time of the laser sensor is about 0.1 ms. The step response time for *x*-stage is *t*_x_ = 1.7 ms; the stable displacement is about *l*_x_ = 12.84 μm; therefore, the response velocity is *v*_x_ = 7.84 × 10^−3^ m/s. The step response time for *y*-stage is around *t*_y_ = 1.6 ms; the stable displacement is about *l*_y_ = 10 μm; therefore, the response velocity is *v*_y_ = 6.25 × 10^−3^ m/s. 

### 3.3. Discussion

Since the designed platform is a 2-DOF platform, this means that there are coupling errors in both *x* and *y*-stages. Generally, if the closed-loop control method is utilized, it is easy to avoid these coupling errors *e*_cx_ and *e*_cy_. However, displacement sensors are needed for the closed-loop control system, which makes the whole system complicated and expensive. In order to get high positioning accuracy with open-loop control system, we have to analyze the motion displacement and the coupling error. For example, the real motion displacement *L*_y_ of *y*-stage contains two parts: one is the displacement *l*_y_ caused by input voltage *V*_y_, the other part is the coupling error *e*_cy_ induced by input voltage *V*_x_. Therefore, the real motion displacement *L*_y_ is achieved by the following:

(28) $$L_{y}=l_{y}+e_{\mathrm{cy}}=-2\times 10^{-6}V_{y}{}^{3}+0.0004V_{y}{}^{2}+0.0889V_{y}-0.0689+0.0045V_{x}+0.0627$$

where the relationship between *l*_y_ and *V*_y_ is obtained from Figure 8a.

**Figure 12:**
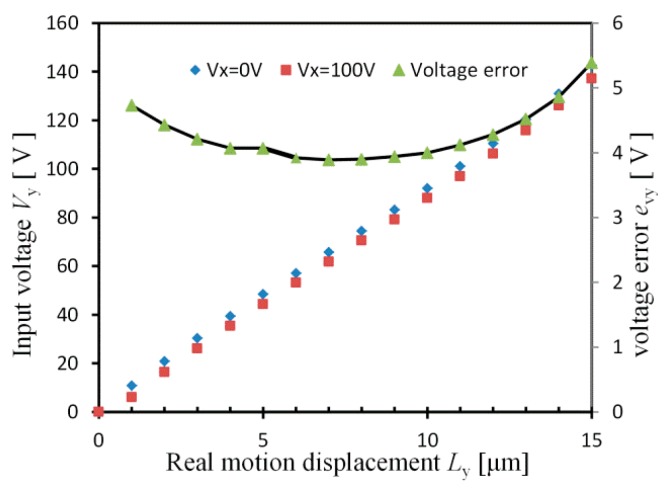
Relationship between the real motion displacement *L*_y_ and the needed input voltage *V*_y_.

In this open-loop control method, three steps are needed to achieve the real motion displacement *L*_y_: firstly, the input voltage *V*_x_ should be known to calculate the error *e*_cy_; next, wanted *l*_y_ is got by using wanted *L*_y_ subtracts the error *e*_cy_; finally, the need *V*_y_ is obtained by Equation (28). Figure 12 shows the needed input voltage *V*_y_ under different input voltage *V*_x_. It is illustrated that, in order to get high accuracy, different *V*_y_ should be given under different *V*_x_. The largest voltage error of the applied *V*_y_ is *e*_vy_ = 5.39 V, in the case that *V*_x_ = 0 V and 100 V. By applying the needed *V*_y_ from the Equation (19), the wanted real motion displacement *L*_y_ is obtained by this open-loop method.

## 4. Conclusions

A compact 2-DOF piezoelectric-driven platform based on “Z-shaped” flexure hinges has been proposed; the experimental system was established to test its performance. Different types of flexure hinges are calculated and compared; and “Z-shaped” flexure hinge was confirmed to have the smallest working stress under the same deformation. MCM and FEM methods were utilized for the optimization of the proposed 2-DOF piezoelectric-driven platform. The maximum motion displacement for *x*-stage is *l*_x_ = 17.65 μm in the case of input voltage *V*_x_ = 150 V; the maximum motion displacement of *y*-stage is *l*_y_ = 15.45 μm in the case of input voltage *V*_y_ = 150 V. The step response time for *x*-stage is about *t*_x_ = 1.7 ms, while that for *y*-stage is *t*_y_ = 1.6 ms. All the experimental data indicate that “Z-shaped” flexure hinges are quite suitable for the design of piezoelectric-driven platforms to obtain submicron accuracy and microsecond response time. This study is meaningful for the application of piezoelectric-driven platform in 3D cellular bio-assembly system. 

## Figures and Tables

**Table 1:** Deformation calculation results of three flexure hinges.

Method	Right-Angle	Right-Circular	Z-Shaped
MCM (Matrix-based compliance modeling)	265.8 μm	28.0 μm	323.5 μm
FEM (Fine element method)	261.4 μm	27.4 μm	340.1 μm
Error	1.7%	2.2%	4.9%

**Table 2:** FEM dynamic modal simulations.

Modal Number	Frequency (Hz)	Resonance Direction
First	678	*y*
Second	965	*z*
Third	1297	*θ*_x_
Fourth	1553	*x*
Fifth	1832	*θ*_z_
Sixth	2002	*θ*_y_
